# Low Complexity Induces Structure in Protein Regions Predicted as Intrinsically Disordered

**DOI:** 10.3390/biom12081098

**Published:** 2022-08-10

**Authors:** Mariane Gonçalves-Kulik, Pablo Mier, Kristina Kastano, Juan Cortés, Pau Bernadó, Friederike Schmid, Miguel A. Andrade-Navarro

**Affiliations:** 1 Institute of Organismic and Molecular Evolution, Faculty of Biology, Johannes Gutenberg University of Mainz, 55128 Mainz, Germany; 2 LAAS-CNRS, Université de Toulouse, CNRS, 31400 Toulouse, France; 3 Centre de Biologie Structurale (CBS), Université de Montpellier INSERM, CNRS, 34090 Montpellier, France; 4 Faculty of Physics, Johannes Gutenberg University of Mainz, 55128 Mainz, Germany

**Keywords:** intrinsically disordered regions, low complexity regions, protein structure, homorepeats

## Abstract

There is increasing evidence that many intrinsically disordered regions (IDRs) in proteins play key functional roles through interactions with other proteins or nucleic acids. These interactions often exhibit a context-dependent structural behavior. We hypothesize that low complexity regions (LCRs), often found within IDRs, could have a role in inducing local structure in IDRs. To test this, we predicted IDRs in the human proteome and analyzed their structures or those of homologous sequences in the Protein Data Bank (PDB). We then identified two types of simple LCRs within IDRs: regions with only one (polyX or homorepeats) or with only two types of amino acids (polyXY). We were able to assign structural information from the PDB more often to these LCRs than to the surrounding IDRs (polyX 61.8% > polyXY 50.5% > IDRs 39.7%). The most frequently observed polyX and polyXY within IDRs contained E (Glu) or G (Gly). Structural analyses of these sequences and of homologs indicate that polyEK regions induce helical conformations, while the other most frequent LCRs induce coil structures. Our work proposes bioinformatics methods to help in the study of the structural behavior of IDRs and provides a solid basis suggesting a structuring role of LCRs within them.

## 1. Introduction

Intrinsically disordered regions in proteins (IDRs) are normally defined as unable to fold into secondary or tertiary structures [1,2,3,4]. Proteins with IDRs are abundant in eukaryotes, where most of them function as interactors of other proteins or nucleic acids [5,6,7]. Despite their inherent lack of structure, it has been proposed that generally IDRs might gain structure upon interaction [8].

The disordered nature of IDRs is defined by their amino acid composition, which is normally enriched in charged and non-structuring residues. Moreover, IDRs display local compositional variations that may be associated with specific functional roles [9]. In previous work, we observed an association of compositionally biased regions within IDRs with their protein interaction sites [10]. We hypothesized that the regions of low complexity frequently found within IDRs could favor structural motifs and facilitate partner recognition. Low complexity regions (LCRs) are protein regions with biased composition, where the amino acid content presents a reduced diversity from the common distribution of amino acids.

About 20% of eukaryotic and 8% of prokaryotic residues in proteins are involved in LCRs [11]. While LCRs have been generally considered to be disordered, they can gain structure depending on their sequence, particularly if they have repeating patterns [11]. An analysis of protein structures in the protein databank (PDB) focusing on LCRs observed that almost 86% present secondary structure preferences, with the majority showing more than one type of secondary structure [12]. Some studies report tracts of repeated single amino acids (homorepeats or polyX) as promoters of well-ordered structures, often helical [13,14,15], with participation of the flanking regions [16]. Furthermore, polyX have been found to be longer in IDR segments than in structured regions [17].

Recent studies focused on the analyses of the general role of LCRs on PDB structures [12,18], while others evaluated different sizes and the diversity of tandem repeats in IDRs [17]. In the present study, we targeted the direct relation between annotated IDRs and simple LCRs. For this, we screened the human disordered proteome and mapped it to high-resolution structures in an attempt to better understand the structural effects of simple LCRs inserted in these IDRs. We targeted LCRs composed of one or two amino acids, here labeled as polyX and polyXY, respectively.

## 2. Materials and Methods

### 2.1. Dataset Construction

**Figure 1:**
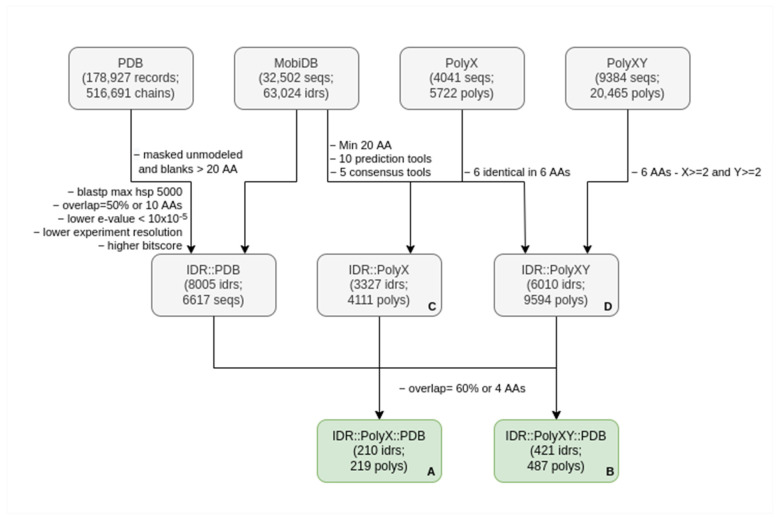
**Description of the process to create the target dataset**. The first number in each box refers to items from the original set from where the second items were extracted, e.g., IDRs from sequences or polyXYs from IDRs, with the exception of the PDB box, which accounts for PDB records and chains. See the Materials and Methods for details. The names used in parentheses indicate: seq, sequences; poly, polyXs or polyXYs; and idr, IDRs. (**A**) Final set of IDRs overlapping PDB sequences that contain polyXs; (**B**) Final set of IDRs overlapping PDB sequences that contain polyXYs; (**C**) Number of IDRs that contain polyXs independently of overlaps with PDB sequences and; (**D**) Number of IDRs that contain polyXYs, also independently of overlaps with PDB sequences.

We obtained the coordinates of all consensus predicted IDRs from MobiDB version 4.0.1, for all the set of human proteins corresponding to the UniProt release 2020_06 (75,796 proteins). MobiDB restricts IDRs to a minimum size of 20 amino acids [19]. The December 2021 version of sequences related to the PDB entries were downloaded for a total of 178,927 PDB records including 516,691 chains (pdbaa; [20]). These and the next steps of the analysis are summarized in Figure 1.

### 2.2. Finding Sequences of Proteins in the PDB with Homology to IDRs

To obtain sequences with known structure homologous to IDRs, BlastP version 2.10 was executed locally with default parameters and limitation of 5000 high-scoring segment pairs (hsp) [21] to compare the 32,502 complete human sequences with IDRs against pdbaa. As pdbaa only provides sequences, without information on which regions are missing from resolved structures, we masked regions without structural information prior to the BlastP search.

To annotate structural information in PDB sequences, we used the dictionary of protein secondary structure (DSSP) [22]. DSSP uses the hydrogen-bonding pattern provided in the 3D files to assign the most likely conformation to each residue of the sequence. Regions of 20 or more consecutive blanks (no structural assignment in DSSP) were masked.

Alignments between PDB sequences and (identical or homologous) IDRs were selected. To obtain the best alignment hit to the IDR, alignments with at least 50% of the IDR region or 10 residues were selected, and only the PDB sequence with the longest alignment, lowest e-value (<10 × 10^−5^) and highest experimental resolution and bit score, was assigned to the corresponding IDR. From the set of 63,024 IDRs from MobiDB, 8005 aligned (totally or partially) with at least one PDB sequence with the e-value < 10 × 10^−5^. 

### 2.3. Secondary Structure Annotation

We considered the DSSP designations to annotate structural information in PDB sequences: (H) for α-helix, (G) for 3/10 helix, (I) for π-helix, (B) for β-bridges, (E) for extended β-strand ladders, (T) for turns, (S) for bends and blanks (“ ”) for residues with low curvature in a not H-bonded structure. Here, we grouped H, G and I as helices; B and E as sheets; and T, S and blanks as coils. Additionally, the missing residues of the PDB structure were masked as (X), and the gaps added by the alignment to the PDB sequence were identified with dashes (-), while the columns of the alignment with gaps in the IDR sequence were removed. 

### 2.4. Filtering PolyXs and PolyXYs Related to IDRs

We identified two types of LCRs, homorepeats (polyX) and polyXY, in all human sequences. PolyX were defined as consecutive stretches of at least six identical residues. PolyXY were defined as regions formed by the overlap of six-residue windows containing only amino acids X or Y, with each of the two types occurring more than once. The threshold of 6 was used following previous work on the length-dependent structural context of polyQ [23]; this threshold is more permissive than the one employed in some general polyX analyses (e.g., eight identical amino acids in a window of 10 residues; [24]). Note that polyX and polyXY can partially overlap.

We selected those LCRs that overlapped with IDRs (at least 60% of the LCR or four residues). Then, we finally selected LCRs if they overlapped the IDR part aligned to a PDB sequence (at least 60% of the LCR or four residues). Two different datasets resulted from this final filtering: 219 polyXs present in 210 IDRs (Supplementary Table S1; A in Figure 1) and 487 polyXYs present in 421 IDRs (B in Figure 1; Supplementary Table S2). More restrictive thresholds for the selection of LCRs strongly impacted the number of cases found (data not shown).

### 2.5. Additional Extractions and Analyses

Searching for additional validation of our results, we submitted the 100-residue fragments surrounding polyXs or polyXYs found in IDRs (C and D in Figure 1, respectively) to the Local Structural Propensity Predictor (LS2P) method [25]. LS2P is based on a statistical analysis of three-residue fragments extracted from SCOPe, a database for protein structural classification [26] and predicts the propensities of IDR sequences to locally adopt secondary-structure-like conformations. As in previous studies [27,28], structural classes were grouped in three different categories: *Helical* comprises all helical structures; *Extended* contains β-strand-type and PPII-type conformations; and *Others* is comprises the remaining mixed structures not classified in the previous categories.

In-house scripts were designed in Python 3.8.10 to extract and transform the outputs of all data sources. The package biopython was used to extract DSSP annotations [29]. Physical-chemical properties of IDRs were calculated with CIDER [30]. Tables and statistical analyses were produced with R 4.1.3 and figures, with ggplot2 version 3.3.5. Protein molecular structures were generated with Chimera 1.15 [31].

## 3. Results and Discussions

Low complexity regions (LCRs) are frequently found within intrinsically disordered regions (IDRs) [10] and can adopt secondary structures [12]. To identify if simple LCRs (polyX and polyXY) have structuring effects on IDRs, we (i) obtained all sequences of IDRs in the human proteome, (ii) identified homologous sequences in the PDB databank of protein structures and (iii) studied the structural information comparing IDRs, polyX and polyXY contained within them (Figure 1; see the Materials and Methods for details).

We found homologous sequences with structure in the PDB for 8005 IDRs in 6617 human proteins (about 13% of the IDRs considered). This covered 164,214 residues out of the 3,833,324 involved in IDRs (about 4%). On the other hand, we found that 3327 and 6010 IDRs had polyX and polyXY, respectively (10% and 18% of the IDRs; C and D in Figure 1).

The datasets of polyX and polyXY in IDRs that overlap with homologous regions in PDB structures contain 219 polyX and 487 polyXY (A and B in Figure 1; Supplementary Table S1 and Supplementary Table S2, respectively), with sizes ranging from 4 to 17 residues and a mean of 7.1 residues in polyXs and sizes from 4 to 18 residues and a mean of 6.9 residues in polyXYs.

We performed a comparison between the set of IDRs containing polyXs and polyXYs with significant and non-significant overlaps with sequences of the PDB structures regarding several canonical IDR characteristics (Supplementary Table S3). We observed that polyXY in IDRs that align to PDB have slightly larger odds of being observed in peripheral regions of the IDR (first or last 12 residues of the IDR or first or last 30% residues of the IDR if the IDR is shorter than 40 residues; odds ratio of 1.61, *p*-value < 0.001). This was not the case for polyX (*p*-value = 0.091). IDRs aligning to PDB were significantly shorter, both for polyX and polyXY sets; however, the LCRs themselves were not significantly different.

When observing some of the canonical characteristics of IDRs [32], we identified slightly lower hydrophobicity and higher fraction of charged residues (FCR) and distributions of oppositely charged residues (*kappa*) in the group that aligns to PDB for polyXs and polyXYs (all values present a Wilcoxon-test *p*-value < 0.001). While a lower *kappa* supports a higher tendency for structural gain in the set of IDRs overlapping PDB, lower hydrophobicity and higher FCR do not. Taken together, these results suggest that the length of the IDR and the position of the LCR inside it might be more relevant than the properties of the IDR in triggering the overlap of the IDR to PDB structures. 

### 3.1. Specific PolyX and PolyXYs Can Produce Structural Gain in IDRs

Considering the 8005 IDR sequences with homology to the PDB (hereinafter, PDB-IDRs), they totaled 413,476 residues, of which 164,124 were covered by homology to PDB structures (40%). This coverage was higher in the polyX present in those IDRs: from 226 polyX (covering 1696 residues), 219 had homology to PDB (covering 1049 residues, 62%). The coverage was also higher for the polyXY in those IDRs: from 605 polyXY (covering 4503 residues), 487 had homology to PDB (covering 2275 residues, a 51%). 

Our results indicate that, in those IDRs with homology to PDB structures, polyX and polyXY had a higher propensity than the background to adopt a secondary structure. PolyXs had a probability of 0.95 of being aligned to a structured residue, while a residue of the IDR that does not belong to a polyX had a probability of 0.489, with a *p*-value < 0.001 on Fisher’s exact test. PolyXYs present a slightly lower probability of 0.948 against 0.488 in non-PolyXY residues (*p*-value < 0.001). Our results suggest that these simple LCRs indeed restrict the inherent flexibility of IDRs (see details in Supplementary Table S4).

It is interesting to note that some amino acids occur more frequently in these LCRs (Table 1 and Table 2; see details in Supplementary Tables S4–S6): glutamic acid (E) stands out as being the most frequent amino acid forming polyX regions and is present in three of the most frequent polyXYs (polyDE, polyEK and polyEP). The role of glutamic acid in IDRs has been already investigated [33]. Glycine (G) is also prominent, ranking second in polyX and present in half of the most frequent polyXYs (polyGS, polyGP and polyGR). Proline (P) ranks sixth as polyX and occurs in two top polyXYs (polyGP and polyEP).

The PDB coverage of these LCRs varies greatly between the different types. Considering the six most frequent polyX in PDB-IDRs (Table 1), polyP has PDB coverage near that of IDRs (40%), whereas polyE has a much higher coverage (68%). For the six most frequent polyXY (Table 2), polyGP stands out with lower coverage than IDRs (30%), while polyEP has the highest coverage (69%). These results suggest that glycine and proline avoid the formation of secondary structures, separately or associated, which is consistent with their known non-structuring properties, while glutamic acid presents a strong structuring role, in agreement with its tendency to be in helical segments [34].

### 3.2. PolyX and PolyXYs Accumulation in PDB-IDRs

To evaluate the types of LCRs that are most frequently found inside IDRs and PDB-IDRs, we compared the frequency in the entire human proteome, in IDRs and in PDB-IDRs of polyX (Table 1; Supplementary Table S5) and polyXY (Table 2; Supplementary Table S6). 

Regarding polyX, we found that polyE, polyG and polyS ranked highly in proteomes, IDRs and PDB-IDRs. PolyD was much better ranked in PDB-IDRs. PolyP ranked low in PDB-IDRs, while being the second most frequent polyX in IDRs and in the proteome. PolyA seems to be rare in IDRs altogether (ranking eighth and seventh in PDB-IDRs and IDRs) although it is the third most frequent polyX in the proteome (see Supplementary Table S5).

Interestingly, polyEK, the second most common polyXY inside PDB-IDRs, is actually not that common inside IDRs or in the complete proteome (10th and 13th in these rankings, respectively). The same can be observed for polyEP and polyGR. 

### 3.3. Secondary Structures from the PDB Associated with PolyX in IDRs

**Figure 2:**
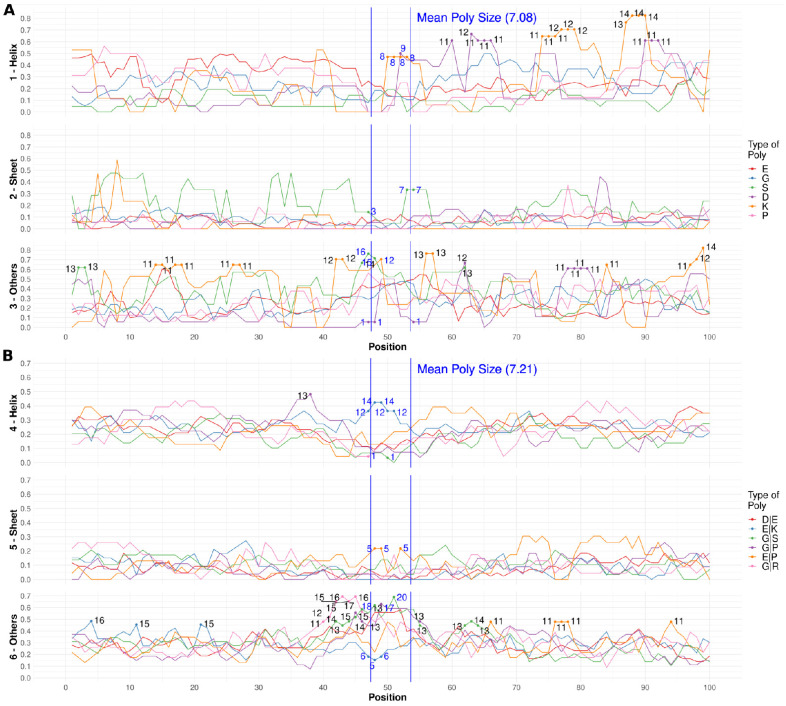
**Secondary structure in PDB homologs for simple LCRs by type**. For each of the six most frequent polyX/polyXY (see legend for type), in a region of 100 residues centered in the polyX/polyXY, fraction of residues in aligned PDB sequences adopting structure. (**A**) polyX (1–Helix, 2–Sheet and 3–Others). (**B**) polyXY (4–Helix, 5–Sheet and 6–Others). See the Methods and Materials for details. The numeric annotations indicate absolute count values at 2% and 98% percentiles to highlight the lower and higher values for each structure type. The blue vertical lines delimit the mean region where polyXs or polyXYs are located.

To understand the role of polyX in structure gain, we studied the types of secondary structure of the PDB sequences aligned to each type of polyX in a region of 100 residues centered at the LCR (Figure 2A). Note that these regions may extend outside the IDRs.

In the interpretation of these data, it is necessary to note that the amount of data points is low (numerical values indicated in Figure 2). In addition, it is possible that multiple cases correspond to proteins of the same family, which can bias the results. To make these issues evident, we included the results for a large region surrounding the polyX (100 residues versus an average size of seven residues). 

Ideally, the surrounding region should indicate the background over which we could observe signal in the middle region indicated for the polyX. A significant signal should look like a large peak (or otherwise a well) in or near the region indicated for the polyX. Otherwise, large peaks in the surrounding region likely mean that the number of cases we are looking at is too low and/or that these cases include multiple homologs that give some signal because they all have a similar structure.

For α-helices, we observe that the counts inside the central polyX region (delimited by vertical blue bars in Figure 2A) present some higher frequencies for polyK and polyD (mainly in the C-terminal region of the polyX) (Figure 2A–Helix). However, the presence of comparable or higher peaks for these polyX outside the LCR suggests that the signal might not be relevant. For β-structures (Figure 2A–Sheet), only polyS presents values near the center of the plot but with other similarly high peaks outside. The most abundant polyE has a wide maximum for coils (Figure 2A–Others). 

Therefore, while polyX appears to induce structure in IDRs, we were not able to assign particular types of secondary structure to given types of polyX, at least with the amount of data available from PDB homologs. However, the cases we collected constitute interesting examples showing that all kinds of secondary structure can be observed in different functional and structural contexts. We illustrate this with a few examples in the following paragraphs (which can be reproduced with the information contained in Supplementary Table S1).

**Figure 3:**
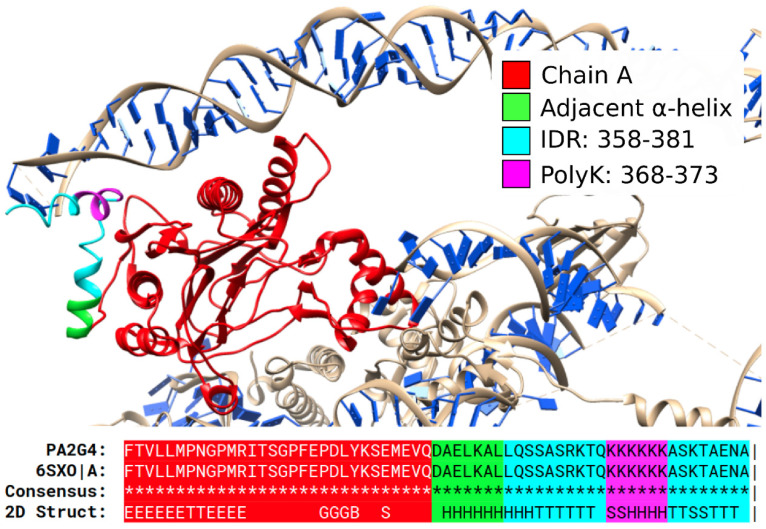
**An α-helical polyK in PA2G4**. Top: PDB:6SXO shows protein PA2G4 (red; UniProt: Q9UQ80) with an IDR containing a polyK with α-helical structure. This conformation could be affected by the folding-on-binding interaction with the 28S ribosomal RNA. Bottom: alignment and structural annotations. IDR and polyK are indicated in cyan and blue. Pipe signs at the end of the alignment indicate that the chain ended at this position.

The structure of the protein PA2G4, solved through electron microscopy (PDB:6SXO), is an example of polyK with α-helical structure (Figure 3). This protein may play a role in the ERBB3-regulated signal transduction pathway, recruiting flexible rRNA and acting as a repressor of the androgen receptor. The predicted IDR region, colored in cyan, starts in the C-terminus of a short helix and becomes a coil. The following polyK, colored in magenta, however, generates a short helix, before another coil region that interacts with a 28S ribosomal RNA. The contact with the RNA sequence in the experiment could cause the observed conformation in a folding-upon-binding interaction [35].

**Figure 4:**
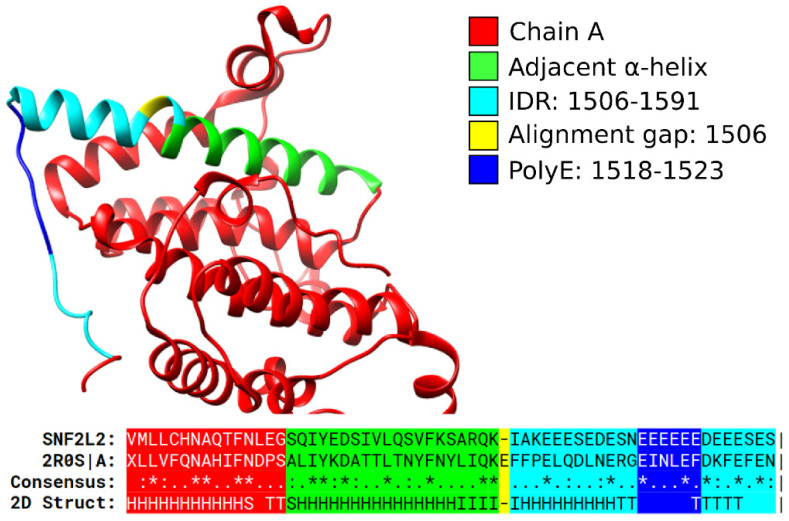
**A polyE in SNF2L2 aligns to a coil region in yeast RSC4**. Structure of the yeast protein RSC4 (PDB:2R0S). Human protein SNF2L2 (UniProt: P51531) with a polyE (blue) inside a predicted IDR (cyan) aligns to RSC4, suggesting that the polyE adopts a coil structure. Pipe signs at the end of the alignment indicate that the chain ended at this position.

Coil structures seem to be commonly induced by all the six most frequent polyX (Figure 2A–Others). As an example, we show here the structure of the yeast protein RSC4, a component of the chromatin structure remodeling complex involved in transcription regulation (PDB:2R0S; Figure 4). The human protein SNF2L2 contains a polyE that aligns to the yeast homolog and could be expected to adopt a similar coil conformation. The AlphaFold model (AF-P51531-F1; [36]) available in the UniProt record of the protein (UniProt: P51531) supports this conclusion.

**Figure 5:**
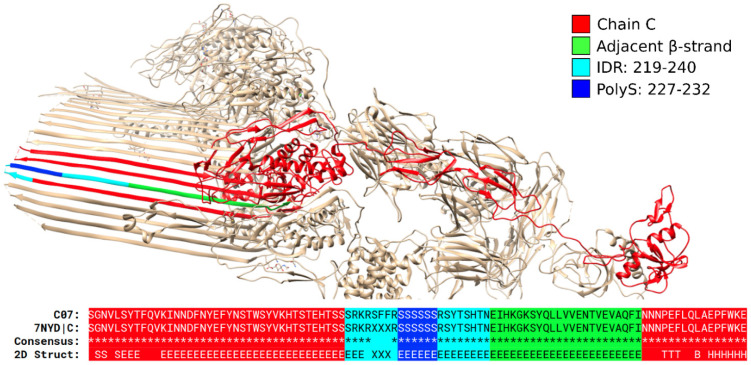
**A polyS in CO7 is part of a β-sheet**. Structure of protein C07 (UniProt: P10643; PDB:7NYD chain C). The polyS (which can be extended according to our definitions to a polyRS region) is part of a long strand of an antiparallel β-sheet.

The values are low for β-sheets, indicating that polyX do not tend to induce this type of secondary structure (Figure 2A–Sheet). As one of the rare examples, we show the structure of protein CO7, complement component C7 (PDB:7NYD chain C; Figure 5). This protein is a constituent of the membrane attack complex MAC, acting as a membrane anchor for the β-barrel structure. When inspected in MobiDB (UniProt: P10643), most of the protein is visualized as structured; however, the region containing the polyS, highlighted in blue, is still predicted as an IDR. Figure 5 shows, however, that the IDR region could be actually much smaller, with the polyS at the beginning of one of the long anti-parallel β-strands.

### 3.4. Secondary Structures from the PDB Associated with PolyXY in IDRs

As above for polyX, to understand the role of polyXY in structure gain, we studied the types of secondary structure of the PDB sequences aligned to each type of polyXY in a region of 100 residues centered at the LCR (Figure 2B). Due to the larger numbers of polyXY cases, more robust conclusions can be extracted.

Again, we need to interpret the data contrasting the values observed in the middle region, expected position of a polyXY with an average size of seven residues (vertical blue lines in Figure 2B) with the entire 100 residue-long region. For helical structures, there is a peak for polyEK, while the other polyXY exhibit a depletion (Figure 2B–Helix). This is suggesting that polyEK induces helical structure in IDRs. For β-structures, lower peaks are observed but they have a similar height than the background peaks (Figure 2B–Sheet). Interestingly, the results for coil structures show as higher than the background peaks inside the central delimited region for polyGS, polyGP and polyDE, with polyGR showing a peak towards the N-terminus. Conversely, polyEK exhibits a depletion of coil structures (Figure 2B–Others). 

Therefore, for polyXY, our analysis associates polyEK with the induction of a helical structure; because of both its peak in α and its depletion in coil conformations (Figure 2B). Most of the other frequent polyXY have a tendency to induce coiled structures (see examples below). As in the previous section, we present a few examples in the following paragraphs (which can be reproduced with the information contained in Supplementary Table S5).

**Figure 6:**
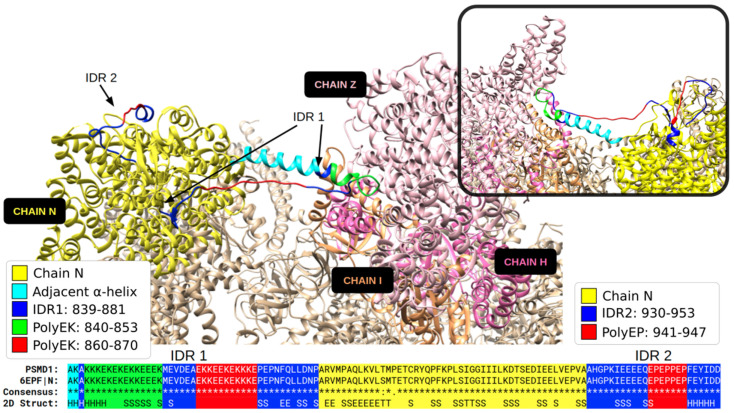
**Two partially structured IDRs in the 26S proteasome non-ATPase regulatory subunit 1**. The human PSDM1 (UniProt: Q99460) aligns to the ortholog in rat (UniProt: O88761). The structure of the sequence from rat (PDB:6EPF chain N) includes one IDR (IDR1) with two polyEK and one IDR (IDR2) with a polyEP. The chains I, H and Z, with which IDR1 interacts, are highlighted. The inset in the upper-right corner shows the rotated superior angle of the structure, focusing on these interactions.

A fascinating example with multiple structured IDRs is the 26S proteasome non-ATPase regulatory subunit 1 protein encoded by PSMD1. The structure of the almost identical rat ortholog suggests that one IDR with two polyEK establishes a contact between two globular domains (Figure 6). It is possible to imagine how this section could be flexible and capable of searching the target structure using a disordered extended loop: when the section would find its target, it would adopt secondary structure, reducing its length, bringing the two domains closer together and stabilizing the overall structure of the complex. Note that, while we assigned a helical structure to polyEK, this example shows that this LCR can adopt helix and coil structures even in the same protein state.

**Figure 7:**
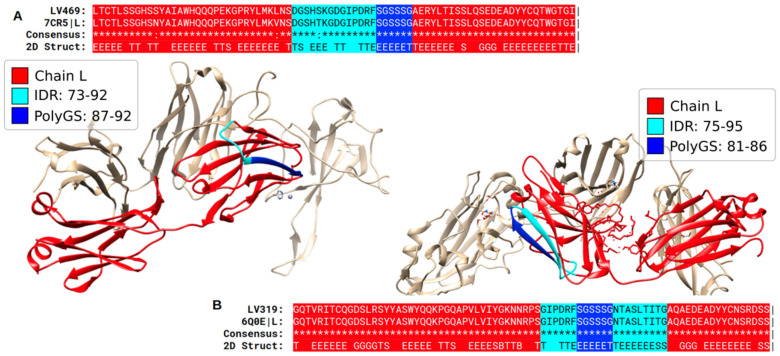
**Structures of immunoglobulin light chains show β structure in predicted IDRs with polyGS**. (**A**) Structure of a human monoclonal antibody (PDB:7CR5 chain L) with a sequence almost identical to protein LV469 Immunoglobulin lambda variable 4–69 (UniProt: A0A075B6H9), a V region of variable domain of immunoglobulin light chains. The polyGS composes one of the strands from one of the four β-sheets of this immunoglobin structure. (**B**) Structure of a human antibody (6Q0E chain L) identical to human protein LV319 Immunoglobulin lambda variable 3–19 (UniProt: P01714). Pipe signs at the end of the alignment indicate that the chain ended at this position.

In addition to helices and coils, polyXY can also form β-structures, albeit more rarely. We show two examples of polyGS in Figure 7 corresponding to immunoglobulin light chains. Here, we might suppose that the region of the predicted IDR will be disordered when the immunoglobulin light chain is in an unbound state and that this structural variability could be helpful to its function of antigen recognition.

### 3.5. Calculation of Structural Propensities for PolyX and PolyXY in IDRs

**Figure 8:**
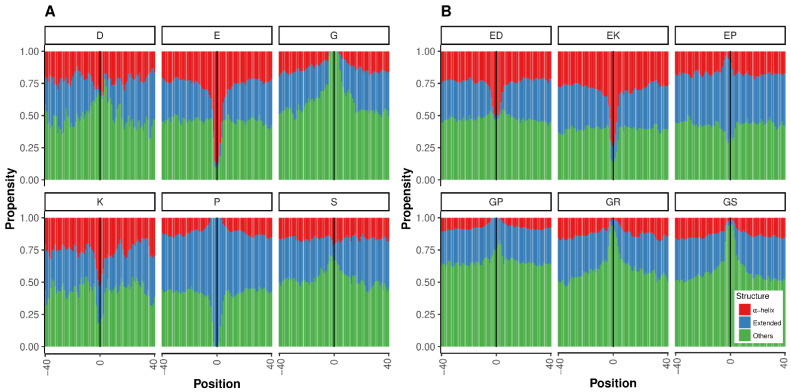
**Structural propensities for simple LCRs in IDRs**. The structural propensities of LCRs and surrounding regions were computed using LS2P (see the Materials and Methods for details). The vertical line indicates the central position of the LCR. (**A**) The top six more common polyX. (**B**) The top six more common polyXY. Structure types shown are helical (red), extended (blue) and others (green).

To complement our study of simple LCR structures within IDRs found in homologs in PDB, we employed an alternative approach that predicts the structural propensities of disordered regions based on statistics obtained from the structures of tripeptides in coil regions from high-resolution structures (LS2P; [25]; see the Materials and Methods for details). For this analysis, we could use all LCRs found in IDRs, even if they were not found in the PDB. For all LCRs considered, there was a difference between the propensities of the background and the LCR, with at least one case among the top six of polyX or polyXY inducing each of three structural states: *Helix*, *Extended* or *Other* (Figure 8). 

Regarding polyX (Figure 8A), polyE stands out as a strong inductor of helical propensity followed by polyK. This result agrees with our findings in the PDB homologs (compare to Figure 2A). PolyP appears to induce extended conformations (experimentally verified in [37]), and coils would be induced by polyG, polyS and polyD (Figure 8A).

In the analysis of polyXY (Figure 8B), the agreement of our results with the PDB homologs is excellent: polyEK is confirmed as a strong inductor of helical propensity (compare to Figure 2B–Helix), polyEP is the only one of the top six showing propensity for extended conformations (compare to Figure 2B–Sheet), and the three glycine-containing polyXY have a tendency to produce coils (compare to Figure 2B–Others). 

For comparison, we computed the values of propensity for synthetic pure LCRs composed of the units displayed in Figure 8, either the polyX or a polyXY made of perfect “XY” repeats. As could be expected, the values for the synthetic polyX were similar to those observed in the center of the graphs for the real shorter LCRs (data not shown). For the sequences made of perfect “XY” repeats, there was mostly good agreement, e.g., the structural propensity of a long “EKEKEKEK” sequence was helical = 0.87, extended = 0.03 and others = 0.11. The largest differences observed were for EP and GP, which had high values of *Extended* (0.94) and *Other* (0.92), respectively, suggesting that the observed polyXY for these categories are rarely made of perfect repeats.

Taken together, these results and those from our study of PDB homologs suggest that K (Lysine) containing LCRs (polyK and polyEK) would have a tendency to induce helical conformations (see examples in Figure 3 and Figure 6), while G (Gly) containing LCRs (polyG; polyGS; polyGP; and polyGR) would have a tendency to produce coil structures. However, extreme variability exists, even within the same LCR type (compare the two polyEK in Figure 6), indicating that structures arising from simple LCRs might have complex dependencies on the sequence and structural context. In relation to context, we note that the background percentage of *Other* is different depending on the polyX and polyXY studied (Figure 8). One would expect same values far away from the LCR. This observation suggests that the composition of the regions surrounding different types of LCR is also different.

## 4. Conclusions

Here, we studied the presence of simple LCRs, polyX and polyXY, within predicted IDRs of the human proteome. We studied their overlap to homologous sequences present in PDB structures. We provide these sequences and homologs as a resource to facilitate the study of IDR structure, dynamics and regulation. Our main observation is that the regions of IDRs containing polyX and polyXY sequences can be associated with structural information from the PDB more often than other parts of IDRs. 

This suggests that the presence of these simple LCRs reduces the conformational flexibility and favors structure. Our analyses indicate that they have different structural propensities than those of the backgrounds and suggest that K (Lys) containing LCRs (polyK and polyEK) induce helicity, while the other most frequent polyXs and polyXYs induce coils. Independently, examination of individual cases indicates a great variation of structures (even for the same polyX or polyXY) and suggests that it might not be possible to assign particular types of secondary structure to particular LCR types.

The examples discussed suggest that predicted IDRs can adopt a structure when establishing interactions with a globular domain (which could be intramolecular or intermolecular) or with RNA/DNA (as in the example shown in Figure 3). This dual ability to remain flexible and to form secondary structures can be helpful to produce conformational changes that can be modulated by interactions and by post-translational modification, thus, giving additional regulatory functions to IDRs containing LCRs. 

Regardless, about two-thirds of the structures used in our analyses involved the protein hosting the LCR in apo-state (see Supplementary Tables S1 and S2, column pdb complex), and therefore the majority of the cases reflect that LCRs in IDRs are able to adopt structure in the absence of intermolecular interactions. We are aware that the accuracy of our alignments between sequences in the PDB and human LCR-IDR-containing sequences without structural information can be affected by the high sequence variability of IDRs. If a particular case needs to be checked, the sequence alignment can be complemented with structure alignment based on a predicted model.

Even with the extension of our study by using homology to sequences in the PDB, our analysis was hampered by the limited number of examples of each type available, which complicates the statistical analyses. While the number of structures in the PDB is increasing, this growth is rather linear, and it is not foreseeable that this situation will be solved in the near future. Probing secondary structural preferences with Nuclear Magnetic Resonance using the recently developed Site-Specific Isotopic Labeling promises to provide key structural information on LCRs [38,39]. 

Alternatively, resourcing to mixed approaches that use information from coil structures in the PDB for structural propensity prediction from sequence (as with LS2P) or conformational studies of small peptides will be likely needed to add more detail to the detection of mechanisms by which IDRs realize rich dynamic structural changes. Recent progress in the use of molecular dynamics simulations to study disordered proteins (e.g., [40]) and, more specifically, LCRs [41] would also be greatly beneficial for the conformational investigation of how LCRs influence IDRs. Despite these limitations and desirable extensions, the work presented herein already provides a methodology and a dataset that can be used to contrast such approaches with the rich structural information of proteins and complexes from all organisms available in the PDB.

## Figures and Tables

**Table 1:** Six most frequent polyXs in IDRs with homology to PDB. See details in Supplementary Tables S4 and S5.

polyX	Count	PDB Coverage	Rank in PDB-IDRs	Rank in IDRs	Rank in Proteome
polyE	91	0.68	1	1	1
polyG	38	0.49	2	4	5
polyS	21	0.65	3	3	4
polyD	18	0.49	4	9	11
polyK	17	0.71	5	6	8
polyP	16	0.40	6	2	2

**Table 2:** The six most frequent polyXYs in IDRs with homology to PDB. See details in Supplementary Tables S4 and S6.

polyXY	Count	PDB Coverage	Rank in PDB-IDRs	Rank in IDRs	Rank in Proteome
polyDE	43	0.57	1	3	4
polyEK	33	0.47	2	13	10
polyGS	29	0.54	3	4	3
polyGP	27	0.30	4	1	2
polyEP	23	0.69	5	18	23
polyGR	23	0.46	6	12	16

## Data Availability

Publicly available datasets were analyzed in this study. This data and the source-code developed to extract and analyze it can be found here: https://github.com/mgkulik/idr-lcr-struct accessed on 5 August 2022.

## Supplementary Materials

The following supporting information can be downloaded at: https://www.mdpi.com/article/10.3390/biom12081098/s1, Table S1: Properties of polyX in IDRs with homology to PDB (set A in Figure 1); Table S2: Properties of polyXY in IDRs with homology to PDB (set B in Figure 1); Table S3: Overall statistical properties of IDRs, polyXs and polyXYs with homology to PDB; Table S4: LCRs and their coverage by homology to PDB; Table S5: Frequency of polyX; Table S6: Frequency of polyXY.
