# Supplementary material for: Low Complexity Induces Structure in Protein Regions Predicted as Intrinsically Disordered
Source: Biomolecules. 2022 Aug 10;12(8):1098. doi: 10.3390/biom12081098 (PMC9405754; doi:10.3390/biom12081098)
Supplement: Supplementary file 1 [file biomolecules-12-01098-s001.zip › Supplementary Tables.pdf]

Supplementary Tables

Supplementary Table S1. Properties of polyX in IDRs with homology to PDB (set A in Figure 1). Columns indicate: UniProt identifier (seq name), identifier of the polyX (poly name), poly size, amino acid type, start and end of the polyX in the query sequence (poly seq start and poly seq end), identifier of the IDR (idr name), size of the IDR (idr size), sequence of the IDR (idr aa), start and end of the IDR in the query sequence (idr seq start and idr seq end), region of the PDB sequence aligned to the IDR (idr pdb), start and end of the region in the PDB sequence aligned to the IDR (idr pdb start and idr pdb end), PDB identifier and chain (pdb name), pdb_complex status (0 = not a complex, PDB file contains one molecule; 1 = complex, PDB file contains more than one molecule), e-value of BlastP (pdb evalue), bitscore of BlastP (pdb bitscore), experiment resolution—when available (pdb resolution), start and end of the region in the PDB sequence aligned to the polyX (poly pdb start and poly pdb end), sequence of the polyX (poly aa), sequence in the PDB sequence aligned to the polyX (poly pdb), secondary structure from DSSP in the PDB aligned to the polyX including blanks (poly ss), 50 amino acid sequence to the left and to the right of the polyX center (poly seq left and poly seq right), 50 amino acid sequence of the aligned PDB sequence to the left and to the right of the polyX center (poly pdb left and poly pdb right) and their secondary structure annotations (poly ss left, poly ss right), UniProt ID of the PDB sequence (aligned id), species of the hit in the PDB (aligned organism). Pipe signs ("|") indicate the end of the sequence range.

Supplementary Table S2. Properties of polyXY in IDRs with homology to PDB (set B in Figure 1). Columns indicate: UniProt identifier (seq name), identifier of the polyXY (poly name), poly size, amino acids XY, start and end of the polyXY in the query sequence (poly seq start, poly seq end), identifier of the IDR (idr name), size of the IDR (idr size), sequence of the IDR (idr aa), start and end of the IDR in the query sequence (idr seq start and idr seq end), region of the PDB sequence aligned to the IDR (idr pdb), start and end of the region in the PDB sequence aligned to the IDR (idr pdb start and idr pdb end), PDB identifier and chain (pdb name), pdb_complex status (0 = not a complex, PDB file contains one molecule; 1 = complex, PDB file contains more than one molecule), e-value of BlastP (pdb evalue), bitscore of BlastP (pdb bitscore), experiment resolution—when available (pdb resolution), start and end of the region in the PDB sequence aligned to the polyXY (poly pdb start and poly pdb end), sequence of the polyXY (poly aa), sequence in the PDB sequence aligned to the polyXY (poly pdb), secondary structure from DSSP in the PDB aligned to the polyXY including blanks (poly ss), 50 amino acid sequence to the left and to the right of the polyXY center (poly seq left, poly seq right), 50 amino acid sequence of the aligned PDB sequence to the left and to the right of the polyXY center (poly pdb left and poly pdb right) and their secondary structure annotations (poly ss left and poly ss right), UniProt ID of the PDB sequence (aligned id), species of the hit in the PDB (aligned organism). Pipe signs ("|") indicate the end of the sequence range.

Supplementary Table S3. Overall statistical properties of IDRs, polyXs and polyXYs with homology to PDB. Columns indicate: Property (column A), group of homology to PDB and statistical results (column B), counts and fractions (Termini poly property) and mean/median (remaining properties) for polyX and polyXYs sets (columns C and D). Property Termini poly describes the LCRs in the termini of IDRs. Termini are defined as first or last 12 residues of the IDR or first or last 30% residues of the IDR if the IDR is shorter than 40 residues. Fisher's exact text was performed in this property, while the Wilcoxon rank test was performed in the remaining properties.

Supplementary Table S4. LCRs and their coverage by homology to PDB. The first column indicates the type of LCR (e.g., polyE, polyDE) or sum of all values for homorepeats or LCRs of two amino acid types (polyX and polyXY, respectively). The following columns indicate for each feature: number of times found in the proteome (n in proteome), number of times found in IDRs and residues covered (n in IDRs, aa in IDRs), number of times found in IDRs with homology to PDB and residues covered (n in PDB-IDRs, aa in PDB-IDRs), number of times found to have homology to PDB and residues with homology (n PDB, aa PDB). The last two columns report the fraction of residues covered by homology in PDB-IDRs and in all IDRs (PDB cover in PDB-IDRs, PDB cover in all IDRs). Compare to the values for the entire IDRs, which are 0.40 and 0.04.

Supplementary Table S5. Frequency of polyX. Columns indicate: type (polyX), counts and ranking of the polyX in PDB-IDRs, IDRs and proteome.

Supplementary Table S6. Frequency of polyXY. Columns indicate: type (polyXY), counts and ranking of the polyXY in PDB-IDRs, IDRs and proteome.
